# The Neuromodulator-Encoding *sadA* Gene Is Widely Distributed in the Human Skin Microbiome

**DOI:** 10.3389/fmicb.2020.573679

**Published:** 2020-12-01

**Authors:** Arif Luqman, Susanne Zabel, Samane Rahmdel, Britta Merz, Nicole Gruenheit, Johannes Harter, Kay Nieselt, Friedrich Götz

**Affiliations:** ^1^Microbial Genetics, University of Tübingen, Tübingen, Germany; ^2^Biology Department, Institut Teknologi Sepuluh Nopember, Surabaya, Indonesia; ^3^Interfaculty Institute for Biomedical Informatics (IBMI), University of Tübingen, Tübingen, Germany; ^4^Department of Food Hygiene and Quality Control, School of Nutrition and Food Sciences, Shiraz University of Medical Sciences, Shiraz, Iran; ^5^CeGaT GmbH, Tübingen, Germany

**Keywords:** metagenomic profiling, microbiota, microbiome, skin, trace amines

## Abstract

Trace amines (TA) are endogenously produced in mammals, have a low concentration in the central nervous system (CNS), but trigger a variety of neurological effects and intervene in host cell communication. It emerged that neurotransmitters and TA are produced also by the microbiota. As it has been shown that TA contribute to wound healing, we examined the skin microbiome of probands using shotgun metagenomics. The phyla Actinobacteria, Proteobacteria, Firmicutes, and Bacteroidetes were predominant. Since SadA is a highly promiscuous TA-producing decarboxylase in Firmicutes, the skin microbiome was specifically examined for the presence of *sadA*-homologous genes. By mapping the reads of certain genes, we found that, although there were less reads mapping to *sadA* than to ubiquitous housekeeping genes (*arcC* and *mutS*), normalized reads counts were still >1000 times higher than those of rare control genes (*icaA*, *icaB*, and *epiA*). At protein sequence level SadA homologs were found in at least 7 phyla: Firmicutes, Actinobacteria, Proteobacteria, Bacteroidetes, Acidobacteria, Chloroflexi, and Cyanobacteria, and in 23 genera of the phylum Firmicutes. A high proportion of the genera that have a SadA homolog belong to the classical skin and intestinal microbiota. The distribution of *sadA* in so many different phyla illustrates the importance of horizontal gene transfer (HGT). We show that the *sadA* gene is widely distributed in the human skin microbiome. When comparing the *sadA* read counts in the probands, there was no correlation between age and gender, but an enormous difference in the *sadA* read counts in the microbiome of the individuals. Since *sadA* is involved in TA synthesis, it is likely that the TA content of the skin is correlated with the amount of TA producing bacteria in the microbiome. In this way, the microbiome-generated TA could influence signal transmission in the epithelial and nervous system.

## Introduction

The term “trace amine” (TA) has been coined in the early 1970s by Alan Boulton and his colleagues to distinguish a group of endogenous vertebrate monoamines from their more abundant structural relatives, the catecholamine and indoleamine neurotransmitters (Boulton, 1974; Gainetdinov et al., 2018). TA are stored in nerve terminals with classical neurotransmitters such as dopamine (DOP), norepinephrine, or serotonin, and are released together with these classical neurotransmitters (Dewar et al., 1988; Premont et al., 2001). Despite their low abundance (Berry, 2004), there is evidence on the crucial physiological roles of TA in the neuromodulation of synaptic transmission in mammalian brains (Burchett and Hicks, 2006; Gainetdinov et al., 2018).

TA are produced by a wide range of organisms from bacteria to plants and vertebrates. In vertebrates, TA can be formed directly by the action of aromatic *L*-amino acid decarboxylase (AADC) on *L*-phenylalanine, *L*-tyrosine, and *L*-tryptophan, (Boulton and Wu, 1972; Saavedra, 1974; Silkaitis and Mosnaim, 1976; Dyck et al., 1983). TA production in bacteria has been mainly studied in food microorganisms, such as enterococci, lactobacilli, streptococci, lactococci, pediococci, and oenococci which represents the main producers of biogenic amines (Marcobal et al., 2006; Irsfeld et al., 2013; Williams et al., 2014; Barbieri et al., 2019).

Although there is some evidence on the low abundance of amine production in food-associated staphylococci (Rahmdel et al., 2018), the investigation of TA production in this genus has provided information about TA formation by the *Staphylococcus* species (Luqman et al., 2018). In this genus only some species are capable of TA production which can mainly be attributed to the presence of the gene *sadA* encoding staphylococcal aromatic amino acid decarboxylase. SadA decarboxylates tryptophan, tyrosine, and phenylalanine to tryptamine (TRY), tyramine (TYM), and phenethylamine (PEA), in a pyridoxalphosphate (PLP)-dependent reaction. It also decarboxylates dihydroxy phenylalanine (L-DOPA) and 5-hydroxytryptophan (5-HTP) to the neurotransmitters DOP and serotonin (Luqman et al., 2018). TA producing staphylococci triggered the internalization into human colon adenocarcinoma cells by activation of the α2-adrenergic receptor (α2-AR) (Luqman et al., 2018, 2019).

A study of the human intestinal microflora revealed that TA-producing staphylococci are present in the majority of human probands, suggesting a selective advantage. Moreover, a *sadA* deletion mutant of the animal pathogen *Staphylococcus pseudintermedius* showed a lower internalization rate than the parent strain in the presence of aromatic amino acids (AAAs). This may reinforce the hypothesis that the excreted TA interfere with host communication to improve the survival and colonization of the bacteria (Luqman et al., 2018, 2019). More recently it has been shown that TA-producing Staphylococcus epidermidis strains expressing SadA are predominant on human skin and that TA accelerate wound healing by antagonizing the β2-adrenergic receptor (β2-AR) in keratinocytes (Luqman et al., 2020b).

In mammalians, TA are synthesized by aromatic *L*-amino acid decarboxylases (AADC; EC 4.1.1.28) (Boulton and Wu, 1972; Snodgrass and Iversen, 1974; Silkaitis and Mosnaim, 1976; Dyck et al., 1983). Although AADC is widely accepted as the vertebrate synthetic enzyme for PEA, TYM, and TRY, the precursor amino acids are in fact extremely poor substrates for AADC (Christenson et al., 1970; Juorio and Yu, 1985; Gainetdinov et al., 2018). This raises the question whether the endogenous synthesis of TA plays a major role at all, or whether the uptake of TA from food and the production of TA by the microbiota are not as such decisive for exerting effects in mammalians. This assumption is supported by the relatively high concentrations of AAAs and TA present on human skin (10 and 5 μg/100 cm^2^, respectively) (Luqman et al., 2020b). Substantial TA concentrations have also been reported in the human gut in the decreasing order of TYM (from 7.6 to 621 μg g^–1^ of stool sample, depending on the subject), DOP, TRY, serotonin, and PEA (Luqman et al., 2018). These relatively high TA levels on the skin and in the intestine indicate that they are not endogenous but can be of microbial origin.

The PLP-dependent Trp decarboxylases of the common gut Firmicutes *Clostridium sporogenes* and *Ruminococcus gnavus* have been enzymatically and structurally characterized (Williams et al., 2014). However, they assumed that such activities are extremely rare in bacteria. On the other hand, the staphylococcal aromatic amino acid decarboxylase, SadA, is a highly promiscuous enzyme. It decarboxylates all AAAs to TAs, and also dihydroxylated phenylalanine and 5-HTP to the neurotransmitters DOP and serotonin (Luqman et al., 2018). SadA producing staphylococci are prevalent in the gut and the human skin (Luqman et al., 2018, 2019, 2020b).

TA are neuromodulators that may have an impact on the well-being of mammals. Therefore, the answer to the question whether SadA is widespread in the human microbiome or whether it is an exotic exception in some Staphylococcus species, is of great importance. The metagenomic profiling of the microbiome allows the analysis of the phylogenetic distribution of individual genes. In this study we investigated the occurrence of SadA homologs in the human skin microbiome. We could show that the microbiome of each volunteer contained *sadA* homologous genes, with large variations from person to person. In addition, SadA homologs are widely distributed throughout almost the entire bacterial kingdom, especially among representatives of the human microbiota, suggesting that the skin microbiota may be able to produce significant amounts of TA to influence host signaling and physiology.

## Materials and Methods

### Skin Swab Sample Collection and Isolation of Genomic DNA

We collected skin swab samples from 27 healthy people (age 14–78; 18 males and 10 females) by swabbing the antecubital fossa using sterile swab sample collectors. The swabs were resuspended in sterile phosphate buffer saline (PBS). We incubated the resuspended skin swabs with lysostaphin for 1 h and isolated genomic DNA using the Quick-DNA Microprep Kit (Zymo Research).

### Metagenomic Analysis

#### Library Preparation and Sequencing

Genomic DNA was quality controlled and then used as input material for the preparation of sequencing libraries. Library preparation was carried out using the Nextera XT DNA Library Preparation Kit (Illumina). After library preparation, all libraries were pooled and sequenced with a read length of 2 × 100 bp on an Ilumina Novaseq^TM^ 6000 system.

#### Taxonomic and Functional Data Analysis

Ten million of the adapter-trimmed raw forward reads were aligned to the RefSeq protein database (version 94) using Diamond in BLASTX mode (Buchfink et al., 2015). Taxonomic placement was performed using the lowest common ancestor (LCA) algorithm implemented in MEGAN6 Ultimate Edition (version 6.15.2) (Huson et al., 2016). Only Taxa with relative sequence abundances above 0.001% were considered. Functional classification was carried out in MEGAN6 Ultimate Edition (version 6.15.2) (Huson et al., 2016) by assigning the reads to KEGG, SEED, VFDB, and Interpro identifiers.

#### Normalized Read Counts

Sequences of the genes [*arcC* (NC_004461.1), *mutS* (NC_004461.1), *sadA* (NP_763667.1), *epiA* (CUY02274.1), *icaA* (AAC06117.1), and *icaB* (AAC06118.1)] were downloaded from the NCBI database. All reads of all samples were mapped against these sequences using BWA (Li and Durbin, 2009) and the number of mapped reads extracted from the resulting samfiles using samtools (Li et al., 2009). Read counts were then normalized for the number of sequenced reads and the length of the respective genes. Therefore, normalized read counts are comparable between samples and between genes.

#### Phylogenetic Analysis of SadA

We performed a phylogenetic analysis of SadA homologs on four different taxonomic rank levels: within the species *S. epidermidis*, the genus *Staphylococcus*, the phylum Firmicutes and within the whole Bacteria domain. Homologs of SadA (WP_014612792) from *S. pseudintermedius* (Luqman et al., 2018) were identified using different flavor of NCBI’s Microbial BLAST and appropriate databases depending on the taxonomic rank the search was restricted to. To find homologs on the strain level within the species *S. epidermidis* tblastn was used and all complete as well as draft genomes of *S. epidermidis* were used as a database. Within the genus *Staphylococcus*, the phylum Firmicutes, and the domain Bacteria homologs of SadA were identified using blastp. The search within the bacterial domain was restricted to proteins of the RefSeq database (O’Leary et al., 2016) due to the large number of hits. Since several species have more than one putative homolog of SadA, we restricted the hits for each species in all blast runs to the best scoring protein. Based on the results of each individual blast run we computed separate phylogenetic trees of the SadA homologs. All best hits of the tblastn run were translated into protein sequences which were subsequently aligned using Clustal Omega (version 1.2.1) (Sievers et al., 2011). Also, the best hits of the three other blast runs were independently aligned using Clustal Omega. Based on the respective resulting multiple sequence alignment, a phylogenetic tree was constructed using maximum likelihood (ML) analysis using RaxML (version8.2.9) (Stamatakis, 2014). The GAMMA Model of rate heterogeneity was used and all model parameters were estimated by RaxML. LG with empirical base frequencies was used as the protein substitution model. Results were assessed using 100 bootstrap replicates. The best tree of each run was visualized using Interactive Tree Of Life (iTOL, version 4.4.2) (Letunic and Bork., 2019).

#### Consensus Sequence Method

The pyridoxal phosphate (PLP)-dependent aspartate aminotransferase superfamily (fold I) domain (accession cd06450) of SadA was selected and its multiple sequence alignment within the genus Staphylococcus was visualized using Jalview 2.11 (Waterhouse et al., 2009).

#### 3D Protein Structure Prediction

We modeled the 3D structure of SadA homologs using SWISS-MODEL (31).

## Results

### Microbiome Analysis of the Human Skin Microbiota

**FIGURE 1:**
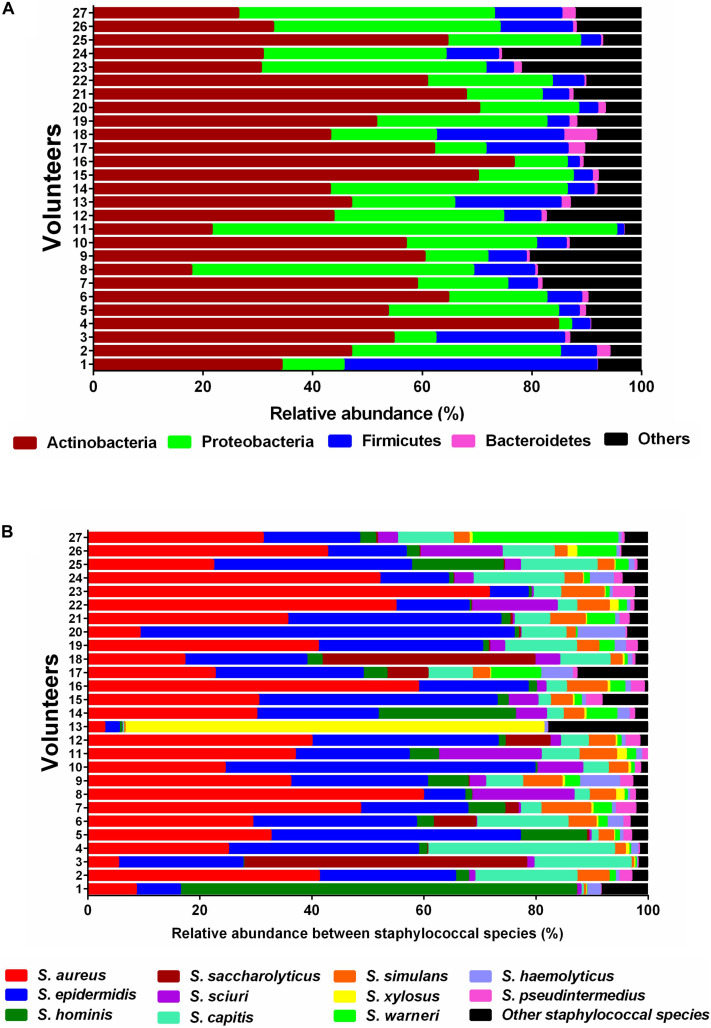
Relative sequence abundances of bacterial phyla and *Staphylococcus* species in skin samples. **(A)** Stacked bar plot of relative sequence abundances of the phyla present in each skin swab sample (*n* = 27). Relative sequence abundances were derived for all phyla present in any sample and the samples sorted by Bray-Curtis similarity. The predominant phyla were Actinobacteria, Proteobacteria, Firmicutes, and Bacteroidetes. **(B)** Stacked bar plot of relative sequence abundances of the species present in each skin swab sample (*n* = 27). Relative abundances were derived for all species present in any sample and the samples sorted by Bray-Curtis similarity. The predominant species were *S. aureus*, *S. epidermidis*, *S. hominis*, *S. saccharolyticus*, *S. sciuri*, and *S. capitis.* The black bars represent the other species with very low relative sequence abundances.

Topographically, the human skin can be divided into many regions and each region represents its own micro-environment (Grice and Segre, 2011). In this study, we performed metagenomic profiling on skin swabs collected from the forearm (antecubital fossa) of 27 probands. This approach enabled us to gain insights into the microbial composition and the phylogenetic distribution of individual genes of the skin microbiome. The most abundant bacterial phyla on the forearm of all probands were Actinobacteria, Proteobacteria, and Firmicutes (Figure 1A). As the genus *Staphylococcus* has been reported to be a very prominent component of the commensal skin microbiota (Kloos, 1980) we specifically focused on the richness and relative sequence abundances of staphylococcal species. In the current study, 28 different staphylococcal species were identified. The most abundant species found in all probands included *S. aureus*, *S. epidermidis*, *S. capitis*, *S. hominis*, and *S. simulans*, followed by *S. saccharolyticus*, *S. haemolyticus*, *S. pseudintermedius*, *S. xylosus*, and *S. warneri* (Figure 1B).

### SadA Homologs Are Widely Distributed in the Human Skin Microbiome

**FIGURE 2:**
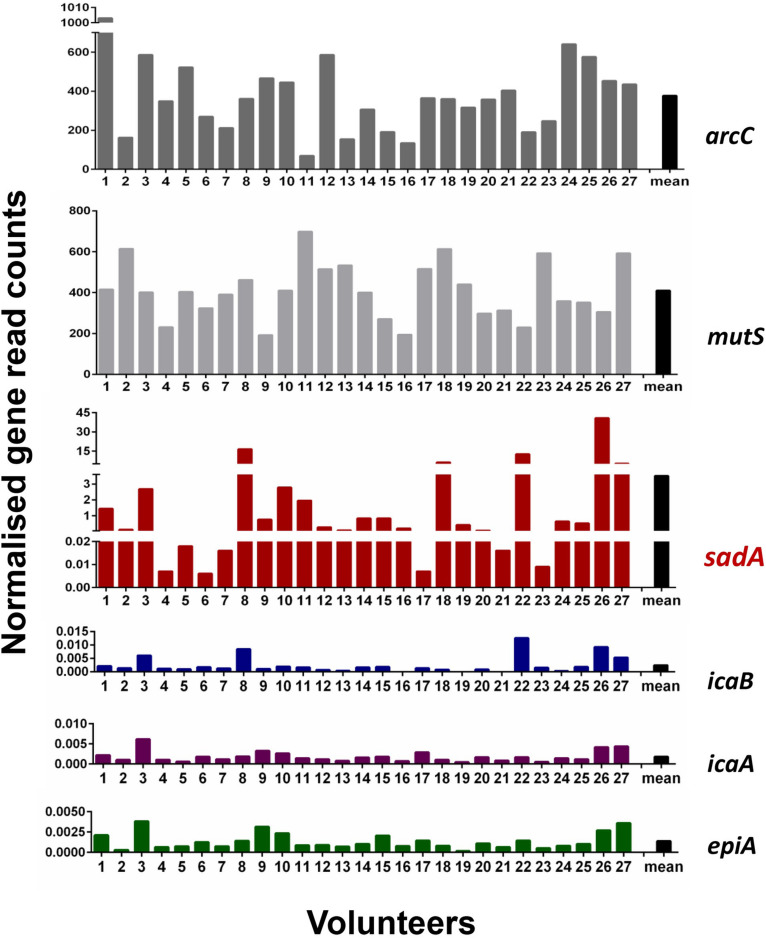
Normalized read counts for *arcC*, *mutS*, *sadA*, *icaB*, *icaA*, and *epiA* genes in skin samples. Reads were mapped against the sequences of the six genes and the obtained read counts normalized for the number of reads per sample and the length of the genes. Paired reads are counted as one.

One of the main goals of this study was to determine how widespread the SadA gene and protein is in the skin microbiome. To investigate the abundance of *sadA* homologs in the skin microbiome, the *sadA* gene sequence from *S. pseudintermedius* (WP_014612792) was used as a reference to analyze the reads obtained from all skin swab samples. As a control, we also determined the normalized read counts of two housekeeping genes *arcC* (Shikimate dehydrogenase) and *mutS* (DNA mismatch repair), which occur almost in all bacteria (Thomas et al., 2007), as well as the less frequently occurring genes *epiA* [representing the structural gene of epidermin biosynthesis (Schnell et al., 1992)] and *icaA* and *icaB* [involved in the biosynthesis of polysaccharide intercellular adhesin (PIA) (Heilmann et al., 1996)]. Normalized read counts of *sadA* were about 400-times lower than those of the housekeeping genes (*arcC* and *mutS*), but more than 1,000-fold higher than the read counts of the rare genes (*icaA*, *icaB*, and *epiA*) (Figure 2). Based on the number of reads mapping to the *sadA* gene, there are more copies of this gene in the skin microbiota than there are copies of the rare genes. Therefore, it is highly likely that there are more bacteria whose genomes contain this gene than the rare genes.

**FIGURE 3:**
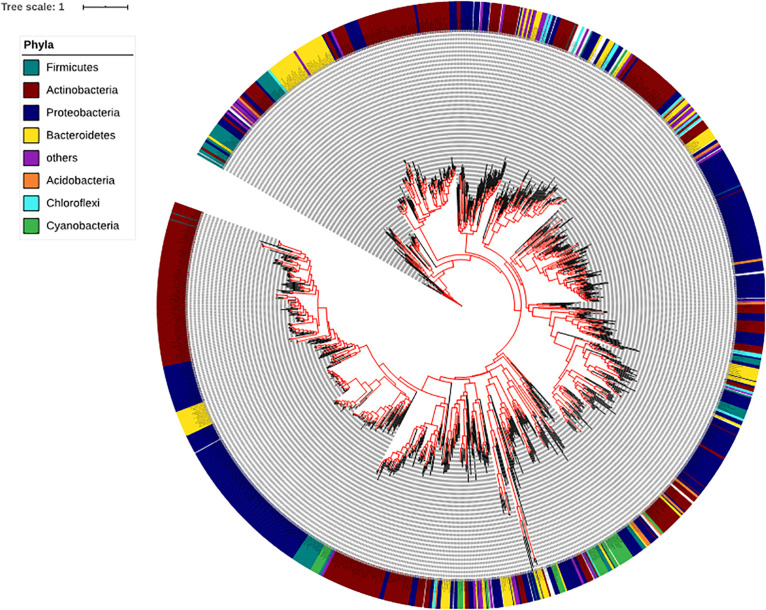
Phylogenetic tree of the bacteria based on the SadA sequence. The SadA sequence from *S. pseudintermedius* ED99 (WP_014612792) was used as a reference to identify homologs within the domain of Bacteria using BLASTP. All best hits were used to construct a phylogenetic tree, using the Randomized Axelerated maximum-likelihood (RaxML) method (version8.2.9) (Sievers et al., 2011). SadA was widespread among bacteria and could be found in at least seven phyla. Based on the SadA sequences, phyla did not cluster perfectly, but the subtrees often consisted of one phylum. The list of bacterial species is shown in Supplementary Table 1.

To determine the presence and absence of the SadA homologs at protein level in all sequenced bacterial genomes we used BLASTP and sequences corresponding to the best hits were then aligned. We then constructed phylogenetic trees based on the alignments. Within the domain of Bacteria, SadA homologs can be found in at least 7 phyla: Firmicutes, Actinobacteria, Proteobacteria, Bacteroidetes, Acidobacteria, Chloroflexi, and Cyanobacteria (Figure 3). This analysis shows that SadA homologs are also widely distributed within bacteria that have been isolated from the skin. In fact, there is a high number of bacterial species that possess SadA homologs (Figure 3 and Supplementary Table 1).

**FIGURE 4:**
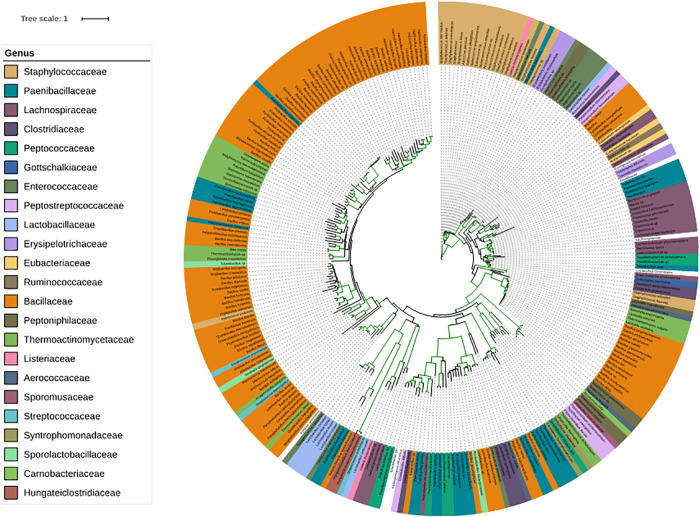
Phylogenetic tree of the SadA sequences within the Firmicutes. The SadA sequence from *S. pseudintermedius* ED99 (WP_014612792) was used as a reference to identify homologs within the Firmicutes phylum using BLASTP. All best hits were used to construct a phylogenetic tree, using the Randomized Axelerated maximum-likelihood (RaxML) method (version8.2.9) (Sievers et al., 2011). SadA was found in many genera of Firmicutes. Based on the SadA sequences, genera were not clustered perfectly, but the subtrees often consisted of one genus. The list of bacterial species is shown in Supplementary Table 2.

Since families belonging to Firmicutes play an important role on our skin, we have given special consideration to corresponding genera. SadA homologs were also found in 23 genera of the phylum Firmicutes (Figure 4), which is comprised of the “classic” low-GC Gram-positive bacteria. Together with the phylum Bacteroidetes, they represent 90% of the gut microbiota (Rinninella et al., 2019). Interestingly, many of the genera that possess SadA homologs like *Clostridiaceae*, *Enterococcaceae*, *Lactobacillaceae*, or *Ruminococcaceae* also belong to the elementary components of the human intestine. (Figure 4 and Supplementary Table 2).

We also analyzed the correlation between the *sadA* read counts and the age of the probands using Pearson correlation; the *p*-value was 0.306. We categorized the *sadA* read counts based on the gender of the subjects and analyzed the difference between the genders using the Student’s *t*-test. This test yielded a *p*-value of 0.868, indicating that intrinsic factors (age and gender) do not show a significant correlation with the *sadA* read counts (Table 1).

**TABLE 1:** *sadA* read counts in correlation with age and gender of probands.

Proband	*sadA* read count	Age	Gender
1	1.438	23	F
2	0.121	29	M
3	2.677	50	M
4	0.007	50	F
5	0.018	28	F
6	0.006	27	M
7	0.016	32	F
8	16.483	32	F
9	0.748	33	M
10	2.78	50	F
11	1.947	36	F
12	0.267	30	M
13	0.072	73	M
14	0.824	34	F
15	0.827	29	M
16	0.19	22	F
17	0.007	30	M
18	6.159	30	M
19	0.41	36	M
20	0.056	31	M
21	0.016	32	F
22	12.493	24	F
23	0.009	29	M
24	0.634	24	M
25	0.515	32	F
26	40.751	24	M
27	5.159	22	F

### The Proposed Active Site Amino Acids of Staphylococcal SadA Are Highly Conserved

**FIGURE 5:**
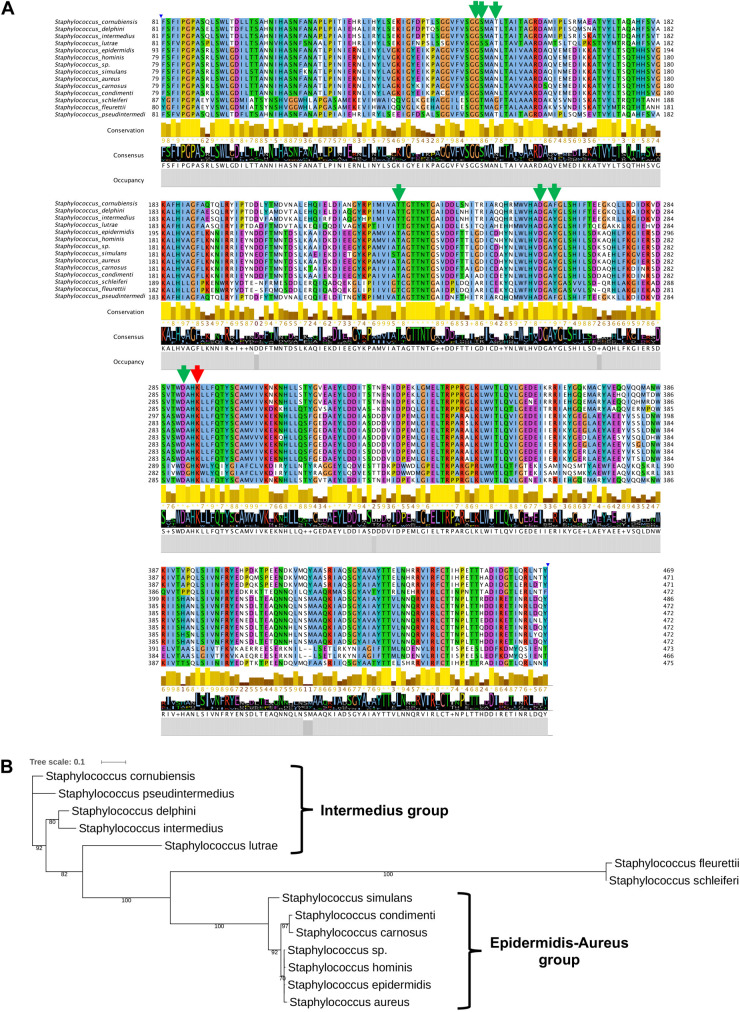
SadA in TA-producing *staphylococci* is highly conserved. **(A)** We aligned multiple sequences of the pyridoxal phosphate (PLP)-dependent aspartate aminotransferase superfamily (fold I) domain (accession cd06450) of SadA from species within the genus *Staphylococcus* and visualized using Jalview 2.11 (Letunic and Bork., 2019). The proposed PLP-binding domain (green) of SadA and the catalytic site Lysine (red) are highly conserved. **(B)** The 475 aa SadA sequence from *S. pseudintermedius* ED99 (WP_014612792) was used as a reference to identify homologs within the *Staphylococcus* genus using BLASTP. All best hits were used to construct the phylogenetic tree, using the Randomized Axelerated maximum-likelihood (RaxML) method (version8.2.9) (Sievers et al., 2011). Node support is indicated by bootstrap values from 100 resamplings of the alignment.

To further substantiate the results, we aligned staphylococcal SadA and marked amino acids (aa) involved in PLP binding and the active site lysine (Figure 5A). The knowledge about the active site aa is derived from structural analysis of Trp decarboxylase (RUMGNA_01526) from *Ruminococcus gnavus*, an anaerobic Gram-positive gut bacterium of the *Clostridiales* family (Williams et al., 2014). This enzyme is 65% similar (44% identity) to SadA, making it currently the most closely related enzyme (Luqman et al., 2018). In all staphylococcal SadA homologs, the pyridoxal-dependent decarboxylase (PLP)-binding domain and the catalytic lysine in the active site were highly conserved. They also had a very similar protein length with about 470 aa.

As shown previously, SadA is not present in all staphylococcal species (Luqman et al., 2018). Using SadA (475 aa) from *S. pseudintermedius* (WP_014612792) as a reference, we constructed the maximum-likelihood phylogenetic tree (Figure 5B). The clustering obtained is broadly consistent with the published phylogram with six species groups and 15 clusters (Lamers et al., 2012). All the species were correctly clustered into the expected groups except *S. schleiferi* which belongs to the intermedius group. *S. fleurettii*, belonging to the oxidase-positive *sciuri* species group is only distantly related with the other staphylococcal species and formed a separate small cluster.

### Confirmation of SadA Homologs in Unrelated Genera

**FIGURE 6:**
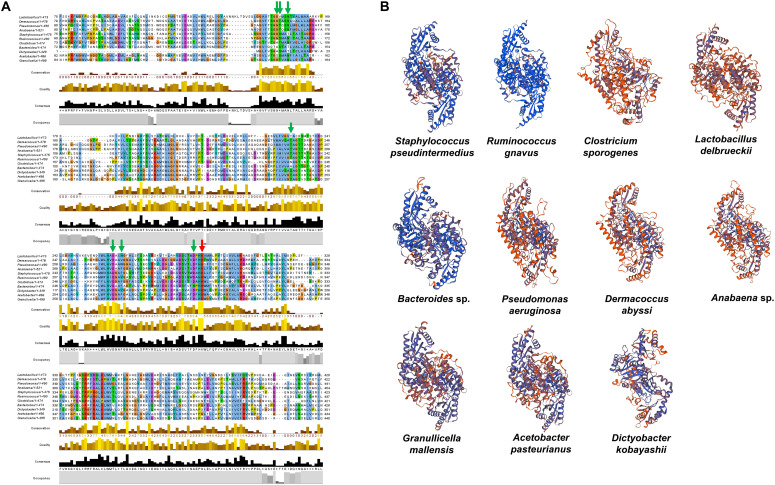
SadA homologs of several bacterial species from different phyla. **(A)** We aligned multiple sequences of SadA homologs of different species from different phyla. Jalview 2.11 was used for visualization (Letunic and Bork., 2019). The PLP-binding motifs are marked with green arrows and the active site lysine with a red arrow. **(B)** We also predicted the 3D structure of SadA homologs using SWISS-MODEL (31). *Staphylococcus pseudintermedius*, *Clostridium spor*ogenes, *Lactobacillus delbrueckii*, and *Ruminococcus gnavus* represent Firmicutes; *Dermacoccus abyssi* represents Actinobacteria; *Pseudomonas aeruginosa* and *Acetobacter pasteurianus* represent Proteobacteria; *Anabaena* sp. represents Cyanobacteria; *Bacteroides* sp. represents Bacteroidetes; *Dictyobacter kobayashii* represents Chloroflexi; and *Granulicella mallensis* represents Acidobacteria. Accession numbers of the SadA homologs used for the alignment: *Staphylococcus pseudintermedius* WP_014612792, *Ruminococcus gnavus* A7B1V0.1, *Clostridium sporogenes* WP_058007894.1, *Lactobacillus delbrueckii* WP_130183735.1, *Pseudomonas aeruginosa* MXH35303.1, *Dermacoccus abyssi* QEH92696.1, *Acetobacter pasteurianus* GAB29948.1, *Bacteroides* sp. KPL16553.1, *Granulicella mallensis* WP_014266912.1, *Dictyobacter kobayashii* GCE20849.1, and *Anabaena* sp. HFS08142.1.

To substantiate the taxonomic presence/absence pattern, we compared SadA with corresponding homologs of representatives of unrelated genera (Figure 6A) and predicted the 3D protein structure of the aa sequences (Figure 6B). The protein length varies from 350–520 aa and the active site amino acid Lysine as well as the proposed PLP-binding sites were highly conserved. The 3D structure models of the homolog proteins show similar folding as SadA from *S. pseudintermedius*. Some of the homologs are annotated as aromatic amino acid decarboxylase, PLP-dependent decarboxylase, or annotated as the domain: Fold Type I–aspartate aminotransferase family with lysine in the active site (Schneider et al., 2000; Milano et al., 2013). The annotation and 3D structure not only confirm the metagenomic analyses but also show that SadA homologs are represented in *Lactobacilli*, *Bacteroides*, *Pseudomonas*, *Acetobacter*, or *Dermacocci*, which all belong to the human microbiota. A comparison of SadA homologs between the genera *Pseudomonas* and *Corynebacterium* showed sequence similarity over most of their length with an average identity of about 27% (Supplementary Table 3).

## Discussion

The promiscuous activity of SadA was the reason why its gene sequence was used to analyze its prevalence in the human skin microbiome. On the human skin, on the forearm (antecubital fossa), the phyla Actinobacteria, Proteobacteria, Firmicutes, and Bacteroidetes predominate (Figure 1A) which is consistent with the previous reports (Grice et al., 2008). Among Firmicutes, staphylococci belong to the classic skin bacteria and play an ambivalent role as typical commensals or opportunistic pathogens. Within this phylum with a focus on the genus *Staphylococcus*, eleven different species were found to be predominant (Figure 1B). Strikingly, *S. aureus* was almost as abundant as *S. epidermidis* in the healthy probands although its primary habitat is the nasal cavity. However, the metagenomic shotgun analysis used (as opposed to a 16S analysis) is able to reliably assign reads down to the species level.

The next question was: to what extent are staphylococcal SadA homologs represented in the entire skin microbiome? Since the gene sequence cannot provide reliable data due to the different GC content in the different phyla, this study was carried out at the protein sequence level. This allowed us to control the active site, the PLP binding site as well as the approximate length of the SadA homologs. We then constructed a phylogenetic tree based on the SadA protein sequence (Figure 3). To verify the metagenomic analyses, SadA homologs from completely unrelated family representatives were aligned and the similarity compared. As shown in Figure 6, the enzymes had a comparable length and the active site amino acids and PLP binding domain were conserved. Therefore, it can be assumed that these are SadA-like decarboxylases. However, nothing can be deduced about their substrate specificity.

Surprisingly, SadA homologs were much more widespread in the bacterial kingdom than expected. SadA homologs were found in at least seven phyla of Gram-positive and -negative bacteria. It is worth mentioning that many families and genera of the Firmicutes, Actinobacteria, Proteobacteria or Bacteroidetes belong to the predominant skin and intestinal microbiota. In depth analysis of the phylum Firmicutes reveals that SadA homologs are represented in 23 genera, many of which belong to the elementary components of the human skin and/or intestine such as *Clostridiaceae*, *Enterococcaceae*, *Lactobacillaceae*, or *Ruminococcaceae* (Figure 4). Interestingly, we did not find SadA homologs in cuti- and propionibacteria, which also belong to the skin microbiota.

From the phylogenetic trees of SadA homologs (Figures 3, 4), one can see that both the phyla and the genus are not perfectly clustered. Furthermore, the subtrees often consist of only one branch and of one genus, which suggests that SadA might undergo lateral gene transfer in the process of evolution. The seemingly arbitrary distribution of *sadA* within a bacterial family suggests horizontal gene transfer (HGT). Since in Gram-positive bacteria phages and prophages are widely distributed phage transduction is the most likely mechanism for lateral *sadA* spreading (Desiere et al., 2002; Novick et al., 2010; Alexeeva et al., 2018; van Zyl et al., 2018). In a metagenomic study on evolutionary trajectory and functional distribution of *S. epidermidis* skin isolates it was reported that they coalesce into multiple founder lineages rather than a single colonizer (Zhou et al., 2020). This is in accordance with our results as only about half of the skin *S. epidermidis* isolates possess the *sadA* gene. However, when we compared the *sadA* synteny in different staphylococcal species, we saw that its position differs within the different staphylococcal species-clusters suggesting transmission via mobile genetic elements (MGEs); but in no case did we see phage-specific flanking genes as relics of a phage transduction, or *sadA* as part of a genomic island (Luqman et al., 2018). Nevertheless, since in Firmicutes and other Gram-positive bacteria phage transduction is more common than conjugation or transformation, we still think that phages play a crucial role in spreading of *sadA*. Perhaps traces of *sadA* acquisition in the genome of staphylococci have become obscured in the course of evolution. However, such traces could possibly be found in other bacteria families.

Our metagenomic analysis represents only a snapshot in the evolution of *sadA*. The question is therefore, is *sadA* in a phase of degression or further expansion. Since *sadA* has no obvious benefit for the growth of bacteria, at least in staphylococci (Luqman et al., 2018), one could imagine a degression of *sadA*. On the other hand, for all bacteria that live together with host organisms *sadA* might be advantageous as it can contribute to colonization and internalization. Given the benefit of *sadA* for host interaction and communication we believe that there is an advantage in obtaining and keeping the gene, at least for such bacterial species whose living is strongly associated with a host; therefore, it is likely that *sadA* spreading is expansive.

What we know so far about SadA and its role for the host is maybe only the tip of the iceberg. We believe that SadA has many other activities regarding the coexistence of bacteria with the host waiting to be revealed. Burchett and Hicks have described TA as “the mysterious TA” (Burchett and Hicks, 2006). In low (“trace”) concentrations, they can interact with TAARs, which play an important role in the coordination of synaptic physiology by potentiating the activity of other neurotransmitters, especially DOP and serotonin. At high concentrations, they have well-characterized presynaptic “amphetamine-like” effects. We show that the intestinal (Luqman et al., 2018) and skin microbiota is quite capable of increasing the concentration of TA, and that the increased TA concentration causes new activities and interactions with receptors. It has been shown already that TA interact with α2- and β-AR and the biological consequences (Luqman et al., 2018, 2019, 2020b). But these are probably not the only receptors with which TA interact. Only recently it has been shown that TA have a high binding activity to the human α_1_-AR and are able to antagonize the epinephrine effect (Luqman et al., 2020a). α_1_-AR is a G protein-coupled receptor in the central and peripheral nervous system; its activation by epinephrine causes among other activities also vasoconstriction. The finding that TA acts as an α_1_-AR antagonist opens completely new perspectives. For example it was found that α_1_-AR antagonists prevent acute respiratory distress syndrome and death from cytokine storm syndrome (Vogelstein et al., 2020), prevent cytokine storm syndrome in COVID-19 (Konig et al., 2020), are used for treatment of lower urinary tract symptoms (Kim et al., 2019), or used for treatments of patients with nightmares after posttraumatic stress disorder and/or borderline personality disorders (Roepke et al., 2017).

When we compare the *sadA* read counts in the probands we see no correlation between age and gender, however, we see an enormous difference in the *sadA* prevalence in the microbiome of the probands (Table 1). Could this difference be related to the unique personality structure and psyche of each individual? The manifold influence of TA on the signaling cascade of the nervous system, the physiology, the behavior, gives an idea of the effects that skin and intestinal microbiota can have on our well-being.

## Data Availability Statement

The original contributions presented in the study are publicly available. This data can be found in the NCBI database using bioproject accession number PRJNA641765. Further information about the associated raw sequence data is shown in Supplementary Table 4.

## Ethics Statement

The studies involving human participants were reviewed and approved by Ethic Commission of the University of Tübingen. The patients/participants provided their written informed consent to participate in this study.

## Author Contributions

FG and AL designed the study. AL, SZ, SR, BM, NG, JH, KN, and FG designed the experiments. AL collected the samples, isolated genomic DNA, and carried out 3D structure modeling. BM, NG, and JH performed the metagenomic analysis. SZ and KN done the phylogenetic analysis. FG and AL wrote the manuscript. All authors contributed to the article and approved the submitted version.

## Conflict of Interest

The authors declare that the research was conducted in the absence of any commercial or financial relationships that could be construed as a potential conflict of interest.

## Supplementary Material

Supplementary Figure 1 Illustration of the sample collection and analysis workflow. We collected skin swab samples from 27 probands, isolated genomic DNA, prepared sequencing libraries and then sequenced these using. The libraries were subjected to next generation sequencing (NGS) analysis.

Supplementary Table 1 List of species in Figure 3.

Supplementary Table 2 List of species in Figure 4.

Supplementary Table 3 SadA homologs found in *Pseudomonas* and *Corynebacterium*.

Supplementary Table 4 Metadata.
