# Supplementary figures and images for: The Neuromodulator-Encoding sadA Gene Is Widely Distributed in the Human Skin Microbiome
Source: Front Microbiol. 2020 Dec 1;11:573679. doi: 10.3389/fmicb.2020.573679 (PMC7736160; doi:10.3389/fmicb.2020.573679)

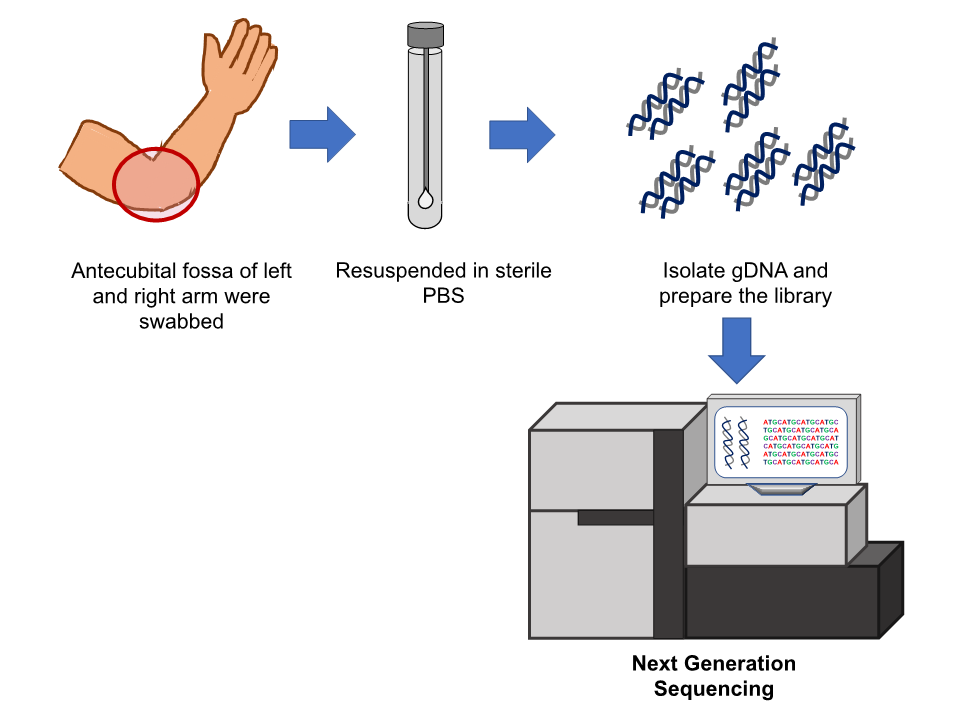

Supplement: Supplementary Figure 1 — Illustration of the sample collection and analysis workflow. We collected skin swab samples from 27 probands, isolated genomic DNA, prepared sequencing libraries and then sequenced these using. The libraries were subjected to next generation sequencing (NGS) analysis. [file Image_1.TIFF]
