# Supplementary material for: The Neuromodulator-Encoding sadA Gene Is Widely Distributed in the Human Skin Microbiome
Source: Front Microbiol. 2020 Dec 1;11:573679. doi: 10.3389/fmicb.2020.573679 (PMC7736160; doi:10.3389/fmicb.2020.573679)
Supplement: Supplementary Table 3 — SadA homologs found in Pseudomonas and Corynebacterium. [file Table_3.docx]

**Supplementary Table 3. SadA homologs found in *Pseudomonas* and *Corynebacterium.***

| **No.** | **Species** | **Protein** | **Query cover (%)** | **Identity (%)** |
| --- | --- | --- | --- | --- |
| 1 | *Pseudomonas donghuensis* | Amino acid decarboxylase | 92 | 27.74 |
| 2 | *Pseudomonas* sp. 250J | Amino acid decarboxylase | 88 | 28.87 |
| 3 | *P. putida* | Amino acid decarboxylase | 87 | 28.24 |
| 4 | *P. putida* F1 | Aromatic L-amino acid decarboxylase | 87 | 28.24 |
| 5 | *P. mossellii* | Amino acid decarboxylase | 88 | 28.87 |
| 6 | *Pseudomonas* sp. Leaf58 | Amino acid decarboxylase | 87 | 28.47 |
| 7 | *P. monteilii* | Amino acid decarboxylase | 87 | 28.94 |
| 8 | *P. entomophila* | Amino acid decarboxylase | 87 | 28.77 |
| 9 | Pseudomonas sp. CCOS 191 | Amino acid decarboxylase | 88 | 28.64 |
| 10 | Pseudomonas sp. 5 | Hypothetical protein | 94 | 26.92 |
| 11 | *Corynebacterium halotolerans* | Pyridoxal-dependent decarboxylase | 80 | 27.34 |
| 12 | *C. macginleyi* | Pyridoxal-dependent decarboxylase | 85 | 25.85 |
| 13 | *C. xerosis* | Aromatic L-amino acid decarboxylase | 84 | 27.72 |
| 14 | *C. heidelbergense* | Pyridoxal-dependent decarboxylase | 68 | 25.51 |
| 15 | *Corynebacterium* sp KPL1856 | Hypothetical protein | 85 | 25.36 |
| 16 | *Corynebacterium* sp HMSC28B08 | Hypothetical protein | 93 | 24.84 |
| 17 | *C. atypicum* | Hypothetical protein | 75 | 26.70 |
